# Associations between Early Markers of Parkinson's Disease and Sarcopenia

**DOI:** 10.3389/fnagi.2017.00053

**Published:** 2017-03-07

**Authors:** Michael Drey, Sandra E. Hasmann, Jan-Peter Krenovsky, Markus A. Hobert, Stefanie Straub, Morad Elshehabi, Anna-Katharina von Thaler, Andreas J. Fallgatter, Gerhard W. Eschweiler, Ulrike Suenkel, Daniela Berg, Walter Maetzler

**Affiliations:** ^1^Medizinische Klinik und Poliklinik IV, Schwerpunkt Akutgeriatrie, Klinikum der Universität MünchenMunich, Germany; ^2^Department of Neurodegeneration, Center for Neurology, Hertie Institute for Clinical Brain Research, University of TuebingenTuebingen, Germany; ^3^German Center for Neurodegenerative Diseases DZNETuebingen, Germany; ^4^Department of Neurology, Christian-Albrechts UniversityKiel, Germany; ^5^Department of Psychiatry and Psychotherapy, University of TuebingenTuebingen, Germany; ^6^Geriatric Center, University of TuebingenTuebingen, Germany

**Keywords:** sarcopenia, neurodegenerative overlap syndrome, prodromal Parkinson's disease, Unified PD Rating Scale, motor unit number index

## Abstract

**Introduction:** Sarcopenia and Parkinson's disease (PD) are both common age-related syndromes, and there is preliminary evidence that the probability of the co-occurrence of these syndromes within one individual is higher than expected. However, it is unclear to date whether one of the syndromes induces the other, or whether there may be common underlying causes. This pilot study thus aimed at investigating the association of the features of increased risk for PD with early stage sarcopenia (ESS).

**Method:** Two hundred and fifty-five community-dwelling individuals were recruited from the *Tübinger evaluation of Risk factors for Early detection of NeuroDegeneration* (TREND) study. The following features that are associated with an increased risk for future PD were evaluated: the motor part of the Unified PD Rating Scale (UPDRS-III), hyperechogenicity of the substantia nigra, prevalence of lifetime depression, hyposmia, REM sleep behavior disorder and the recently introduced probability score for prodromal PD. Sarcopenia was defined according to the European Working Group on Sarcopenia in Older People, which was adapted to this cohort of healthy adults. Multiple linear regression analysis was used to identify associations of PD-related features with ESS.

**Results:** The UPDRS-III score was significantly associated with ESS. The result remained significant after the adjustment for age, gender and physical activity. No association was found between the other PD-related features and ESS.

**Conclusion:** The significant association of the UPDRS-III score with ESS in this cohort might indicate a common and early pathway in both diseases and supports the existence of an “extended neurodegenerative overlap syndrome.” Moreover, the potential of EES to serve as a prodromal marker of PD should be evaluated in future studies.

## Introduction

Parkinson's disease (PD) is the second most common neurodegenerative disease and is characterized, among other symptoms, by bradykinesia, rigidity, tremor and postural instability. The social and economic burden of this age-related disease is high, and the etiology and pathogenesis are only partly understood. In addition to age, male gender, a positive family history for PD and a hyperechogenicity of the substantia nigra (SN+) are risk markers of the disease. Moreover, motor and non-motor prodromal markers occur regularly in PD. In more detail, PD encompasses a prodromal phase of many years or even decades (Gaenslen et al., 2011). During this prodromal phase, there is an increased probability of the occurrence of non-motor features, such as hyposmia, minor and major depression and REM sleep behavior disorder (RBD). During the final years prior to the conversion to clinically overt PD, also subtle motor features not sufficient for diagnosis but still suggestive for the disease are more common in the prodromal phase of PD than in the older population without PD (Schrag et al., 2015). This has been shown for distal as well as for axial motor symptoms such as gait and balance (Maetzler and Hausdorff, 2012). This increased probability of the occurrence of PD-related subtle motor symptoms in the prodromal phase of the disease has also been demonstrated with the use of the motor part of the Unified PD Rating Scale (UPDRS-III) in a longitudinal study (Postuma et al., 2012). The Movement Disorder Society (MDS) has recently introduced a probability score for prodromal PD by considering all currently known risk and prodromal markers of PD. This score basically reflects the likelihood of prodromal PD being present (Berg et al., 2015).

Sarcopenia, defined as age-related loss of muscle mass and function, is also increasingly realized as a serious medical and economic problem in our aging societies (Cruz-Jentoft et al., 2010). Sarcopenia is evident in approximately 20% of individuals over 70 years old; this figure rises to 50% in the over-80 age group (Cruz-Jentoft et al., 2010). The pathways that lead to sarcopenia have not yet been identified. The prevailing concept for the development of sarcopenia is multifactorial (Cruz-Jentoft et al., 2010). Previous investigations have identified a relationship between a low number of motor neurons, as measured with the motor unit number index (MUNIX), and sarcopenia (Drey et al., 2014). Thus, neurodegeneration appears to play a role in the onset of sarcopenia.

There is some evidence that at least a proportion of PD and sarcopenia patients share a common pathway of dopaminergic dysfunction and reduced numbers of motor neurons. Caviness and co-authors have found significantly lower motor unit numbers in the hand muscles of PD patients compared to healthy controls using different MUNE (Motor unit number estimation) techniques, a measure that is comparable to MUNIX (Caviness et al., 2002). In summary, these findings suggest that a common pathway exists at least in subpopulations of both diseases.

We followed this hypothesis by comparing respective parameters in a random subsample of participants of the TREND study (Tübinger evaluation of Risk factors for Early detection of NeuroDegeneration), a cohort of individuals with and without widely accepted risk factors for neurodegeneration.

## Methods

### Participants

For this analysis, a random sample of 255 individuals who were recruited during the second follow-up of the TREND study were assessed with routine follow-up assessments and Bioelectrical Impedance Analysis (BIA). TREND is designed as a prospective biannual longitudinal study that aims to define screening batteries for an earlier diagnosis of PD and Alzheimer's disease (http://www.trend-studie.de). The ethical committee of the Medical Faculty of Tuebingen approved the study protocol, and written informed consent was obtained from all participants.

### Measured values

#### PD-associated measurements

SN+ was measured via transcranial ultrasound. A value of ≥0.22 mm^2^ was defined as hyperechogenicity. Olfaction was investigated using the 16-item Sniffin' sticks (Burghart Medizintechnik, Germany) identification battery with normative age-dependent cut-off values. Individuals below the 10th percentile were classified as hyposmic. Depression was defined by either a positive history of lifetime depressive episode or a value of ≥18 points in the actual assessment of the Becks Depression Inventory (BDI). RBD was diagnosed if the criteria of the International Classification for Sleep Disorders (ICSD) were met. The symptoms were assessed with the RBD Screening Questionnaire (RBDSQ). The overall probability of being in a prodromal PD phase was assessed with a score recently introduced by the MDS, including in the calculation the following features: age, gender, smoking history, positive family history for PD, SN+, RBD, new version of the UPDRS-III (MDS UPDRS-III) score >3 excluding action tremor, hyposmia, constipation, symptomatic hypotension, severe erectile dysfunction, urinary dysfunction and depression (Berg et al., 2015). Motor features were measured with the MDS UPDRS-III score alone. The scale consists of 18 items that rate PD-specific motor features, such as rigidity, tremor, axial and distal bradykinesia, facial expression, and postural deficits. Each item has a score from 0 to 4. It should be noted that this test does not include muscle strength and muscle mass assessments and thus does not overlap with the sarcopenia assessment.

#### Sarcopenia-associated measurements

Muscle mass was calculated using Janssen's regression formula by applying BIA (Janssen et al., 2000). Isometric hand grip strength was determined by using a hand-held dynamometer (JAMAR, USA). Gait speed was measured by recording the time that was required for participants to walk along a 20 m hallway with a stop watch. Physical activity was assessed by asking participants for the duration of sports activity in hours per week. Activity was scaled from “no sport = 5” till “more than 4 h per week = 1.” As the TREND cohort is basically a relatively “young” and population-based cohort, the usual sarcopenia criteria could not be applied due to a floor effect. Therefore, we defined early stage sarcopenia (ESS) in our cohort in participants who exhibited muscle mass index (kg/m^2^), grip strength (kg), and gait speed (m/s) values that were in the lower half of the scales. According to the European Working Group on Sarcopenia in Older People (EWGSOP) criteria (Cruz-Jentoft et al., 2010), the combination of a low muscle mass index AND a low grip strength OR a low gait speed led to classification of ESS. Thirty-eight percent of participants were accordingly classified as having ESS.

### Statistics

PASW 23.0 (IBM-SPSS Inc., Chicago, Il, USA) was used for statistical analysis. The participants' characteristics were expressed as the mean, standard deviation and range of values (Table 1). Multiple linear regression analysis was used to identify the influence of UPDRS-III, SN+, hyposmia, depression, RBD and the probability score for prodromal PD on ESS. Models were adjusted for age, gender and physical activity. The level of significance was set at 5%.

**Table 1:** **Participant's characteristics**.

	**Male (*n* = 150)**	**Female (*n* = 150)**	**All**	***p*-value^a^**
Age [years]	65.1 ± 5.4	64.7 ± 6.6	64.9 ± 5.9	0.611
Gender [%]	59	41	100	0.005^b^
UPDRS-III [p]	1.3[0–29]	0.9[0–20]	1.1[0–29]	0.290
SN positive [%]	24.0	9.5	18.0	0.010^b^
RBD [%]	8.0	8.6	8.2	0.870^b^
Depression [%]	13.3	31.4	20.8	0.000^b^
Hyposmia [%]	38.0	25.7	33.3	0.037^b^
PS for prodromal PD	4.9[0.03–83.2]	1.3[0.02–10.7]	3.4[0.02–83.2]	0.001
Gripforce [kg]	43 ± 7.0	26 ± 5.5	36.0 ± 10.7	0.000
Gait speed [m/s]	1.4 ± 0.2	1.3 ± 0.1	1.4 ± 0.2	0.000
Muscle mass index [kg/m^2^]	10 ± 1.0	8 ± 0.9	9 ± 1.7	0.000
Body Mass Index [kg/m^2^]	28 ± 3.9	27 ± 5.1	28 ± 4.4	0.561
Physical activity score [p]	2.5 ± 1.3	2.4 ± 1.1	2.5 ± 1.2	0.934^c^

*Values show mean and SD/range where appropriate. PS for prodromal PD: Probability score for prodromal PD*.

a *T-test for independent samples*.

b *Chi-square test*.

c *Mann-Whitney U Test*.

## Results

Characteristics of participants are shown in Table 1. In our sample, more male (59%) than female participants were recruited. The mean MDS UPDRS-III values were approximately one point. The frequency of SN+ was approximately 2.5 times higher in males (24%) than in females (10%). A similar distribution was observed for hyposmia (38% vs. 26%). The frequency of depression in females (31%) was almost three times higher than in males (13%). As expected, mean grip force and mean muscle mass index were lower in females (26 kg, 8 kg/m^2^) than in males (43 kg, 10 kg/m^2^).

Results from the logistic regression are shown in Table 2. The MDS UPDRS-III score was significantly associated with ESS. The result remained significant after the adjustment for age, gender and physical activity. No significant association was found between ESS and the following parameters: SN+, hyposmia, depression, RBD and probability score for prodromal PD.

**Table 2:** **Logistic regression with ESS as dependent variable**.

	**Model 1**		**Model 2**		**Model 3**	
	**Odds ratio**	***p*-value**	**Odds ratio**	***p*-value**	**Odds ratio**	***p*-value**
UPDRS-III	2.609 [1.223; 5.563]	0.013	2.273 [1.046; 4.938]	0.038	2.309 [10.058; 5.037]	0.035
SN positive	0.871 [0.447; 1.698]	0.686	0.792 [0.395; 1.590]	0.512	0.795 [0.395; 1.600]	0.520
RBD	0.964 [0.384; 2.418]	0.938	0.969 [0.381; 2.464]	0.948	0.973 [0.382; 2.477]	0.954
Depression	1.040 [0.559; 1.933]	0.902	0.981 [0.511; 1.883]	0.955	0.986 [0.513; 1.894]	0.966
Hyposmia	0.734 [0.425; 1.266]	0.266	0.588 [0.328; 1.053]	0.074	0.608 [0.339; 1.092]	0.096
PS for prodromal PD	1.012 [0.984; 1.040]	0.397	1.000 [0.970; 1.030]	0.989	1.000 [0.970; 1.031]	0.997

*UPDRS-III: Unified Parkinson's disease rating scale, SN: Substantia nigra, RBD: REM-sleep behaviour disorder, PS for prodromal PD: Probability score for prodromal PD. Model 1: unadjusted, Model 2: adjusted for age and gender, Model 3: adjusted for age, gender and physical activity*.

## Discussion

The main finding of this study is that in this basically community-dwelling cohort aged approximately 65 years old with an increased prevalence of people with risk factors for PD but no clinical signs of PD, ESS was associated with subtle motor features that were detectable with the MDS UPDRS-III. The result indicates that ESS is associated with subtle motor deficits in prodromal PD in at least a proportion of individuals.

**Figure 1:**
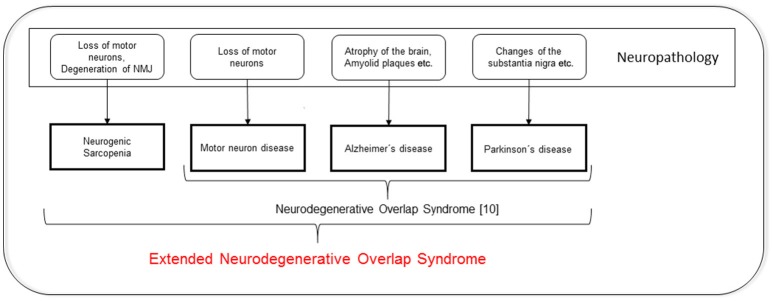
**Extended neurodegenerative overlap syndrome**. NMJ: neuromuscular junction.

To date, neurodegenerative diseases are mainly defined by the dysfunction of relatively circumscribed neuronal systems. For example, for PD, it is mainly the nigrostriatal degeneration that causes motor dysfunction, and for motor neuron disease (MND), it is the degeneration of the upper and lower motor neurons that causes weakness. Most of the neurodegenerative diseases are, in addition to exhibiting the “typical” features, characterized by a loss of muscle mass and a decrease in functional performance. This association makes it tempting to speculate about overlaps between neurodegeneration and sarcopenia. In fact, recent investigations have identified a relationship between low numbers of motor neurons and sarcopenia (Drey et al., 2014), and suggest the presence of neurodegenerative components that lead to sarcopenia. Interestingly, Uitti and colleagues introduced the term “neurodegenerative overlap syndrome,” which includes PD, MND, and even dementia, more than 20 years ago (Uitti et al., 1995). In conjunction with our data, the existence of an “extended neurodegenerative overlap syndrome” that also comprises sarcopenia should be considered. This hypothesis is visualized in Figure 1.

The significant and exclusive association of the MDS UPDRS-III score with the ESS indicates a relation of ESS with late phases of prodromal PD. As shown in previous studies, motor deficits present relatively close to clinical diagnosis (Maetzler and Hausdorff, 2012; Schrag et al., 2015). This late occurrence of motor features is different to most of the non-motor features, which can precede clinical PD by more than a decade. The lack of a relevant association between the ESS and prodromal PD probability scores indirectly supports this conclusion, as only one 10th of the markers defining the prodromal PD probability score refer to motor deficits (Berg et al., 2015).

This link between PD and sarcopenia in their earliest stages as observed here could be driven by neuroinflammation. Elevated levels of circulating inflammatory mediators are detectable in patients in the early stages of both PD and sarcopenia, with overlap in at least some of the species (Scalzo et al., 2010). An example is IL6. This cytokine has been associated with the loss of muscle mass and poor physical performance in older adults (Cesari et al., 2004) and in PD (Scalzo et al., 2010). Another driver could be the increased energy expenditure in PD due to muscular rigidity and tremor. A significantly lower fat-free mass, but a similar resting metabolic rate compared to controls, was found in PD patients, which suggests the presence of a hypermetabolic state in the latter cohort (Poehlman et al., 1995).

This study faces some limitations. First, we are not able to determine whether further, not yet considered parameters could explain our finding. For example, it could be that physical inactivity is at least indirectly (e.g., through the metabolic syndrome) related to the development of PD (LaHue et al., 2016), and physical inactivity is certainly associated with lower muscle mass. Although we included physical activity as a covariable in our model, future studies should investigate such interactions in more detail and with quantitative measures. A second limitation could be the method that was used to estimate muscle mass. Dual energy x-ray absorptiometry (DXA) is considered to be the gold standard for that approach. Nevertheless, the BIA that was used in our study appears to be an adequate alternative. Method comparisons between BIA and DXA to determine muscle mass showed a good correlation. Based on this, muscle mass that was calculated using Janssen's regression formula exhibited a strong correlation (*r* = 0.906) with the muscle mass that was determined by DXA (Bosaeus et al., 2014).

## Conclusion

The association between MDS UPDRS-III and ESS in this cohort with increased PD risk suggests a common pathway during preclinical stages of both diseases. Moreover, EES may be a prodromal marker of PD.

## Author contributions

MD, SH, US, and WM made substantial contributions to the acquisition, analysis and interpretation of data for the work. JK was responsible for data analysis. MD, SH, and WM drafted the paper; all of the remaining authors revised the draft critically for important intellectual content. All of the authors gave their final approval of the version to be published and agreed to be accountable for all aspects of the work to ensure that questions related to the accuracy or integrity of any part of the work are appropriately investigated and resolved.

### Conflict of interest statement

The authors declare that the research was conducted in the absence of any commercial or financial relationships that could be construed as a potential conflict of interest.
